# Socioeconomic inequalities in waist circumference and its 12-year change: Moderation by neighbourhood population density

**DOI:** 10.1016/j.pmedr.2025.103236

**Published:** 2025-09-11

**Authors:** Sungkavi Selvakumaran, Manoj Chandrabose, Nyssa Hadgraft, Neville Owen, Takemi Sugiyama

**Affiliations:** a Swinburne University of Technology, Melbourne, Victoria 3122, Australia; b Centre for Urban Research, RMIT University, Melbourne, VIC, Australia; c School of Health Sciences, Swinburne University of Technology, Melbourne, Victoria 3122, Australia; d Baker Heart & Diabetes Institute, PO Box 6492, Melbourne, Victoria 3004, Australia

**Keywords:** deprivation, health disparities, built environment, interaction, socioeconomic status, obesity

## Abstract

**Objective:**

Reducing socioeconomic inequalities in obesity is a public health priority. We examined whether population density, a key environmental characteristic associated with obesity, moderates the associations of area-level socioeconomic status (SES) with waist circumference and its rate of change.

**Methods:**

Data from adults in an Australian national study, collected across three waves (1999–2000, 2005–06, 2011–12), were used. Outcomes were objectively measured baseline waist circumference (*N* = 4835) and its rate of change (*N* = 1158). Exposure was baseline area-level SES. Baseline population density was calculated within a 1 km buffer around residences. Multilevel regression models were used.

**Results:**

Higher area-level SES was associated with smaller baseline waist circumference (−1.45, 95 % CI [−2.28, −0.12]) but not with change over time (0.04, 95 % CI [−0.02, 0.09]). In stratified cross-sectional analyses, this association was significant in lower- (−5.00, 95 % CI -7.73, −2.28]) and medium-density (−2.61, 95 % CI [−4.55, −0.67]), but not in higher-density areas (−0.54, 95 % CI [−1.73,0.65]). In longitudinal analyses, lower SES was linked to smaller increases in waist circumference in lower- (0.16, 95 % CI [0.03, 0.29]) and medium-density (0.17, 95 % CI [0.05, 0.30]) areas, suggesting a narrowing of socioeconomic inequalities in obesity. However, annual changes were modest and unlikely to offset baseline inequalities.

**Conclusions:**

Population density moderated associations of area-level SES with waist circumference and its change. Inequalities in baseline obesity were pronounced in lower density areas but not higher density areas. While the longitudinal findings of the moderation patterns were different from baseline results, they suggest persistent socioeconomic inequalities in obesity in lower density areas.

## Highlights

• Socioeconomic inequalities in waist circumference differed by population density.
• Baseline inequalities were found in areas of lower density but not of higher density.
• Waist circumference changes were small and unlikely to shift baseline gaps.

## 1 Introduction

Overweight and obesity are associated with a higher risk of non-communicable diseases such as cardiovascular disease, type 2 diabetes mellitus and cancers as well as psychological distress and reduced quality of life (Finer, 2015). In 2021, approximately 1 billion males and 1.1 billion females had overweight and obesity (Global Burden of Disease 2021 Adult BMI Collaborators, 2025). Compared with 1990, the global prevalence of obesity had increased by 155 % in males and 105 % in females, with trends expected to continue without effective interventions (Global Burden of Disease 2021 Adult BMI Collaborators, 2025). Reducing overweight and obesity (referred collectively as obesity) is a public health priority, as the burden of obesity extends beyond individual health and has broader consequences for health systems and economies (Mehrzad, 2020).

Social determinants of health, which include contextual factors of where people live, are considered underlying drivers of obesity, and addressing them is essential for obesity prevention (Biermann et al., 2021). One of the key social determinants of health is the socioeconomic status (SES) of the neighbourhood in which people live (Marmot and Bell, 2016). There are significant inequalities in obesity risk between areas with differing levels of SES (Drewnowski et al., 2014; de Mestral et al., 2024). A meta-analysis, involving 21 observational studies from high-income countries from North America, Europe and Australasia (*n* = 1,244,438), found that residents of lower-SES areas had 31 % higher odds of being overweight and 45 % higher odds of obesity than those of higher-SES areas (Mohammed et al., 2019). The socioeconomic gap in obesity also appears to be widening. A study in Australia found that residents in the lowest SES neighbourhoods had a 2.1 kg/m^2^ greater increase in BMI compared to those in higher-SES neighbourhoods over six years (Feng and Wilson, 2015). A US study also found that residents in lower-SES areas had approximately 6 kg greater weight gain over seven years compared to those in higher-SES areas (Powell-Wiley et al., 2014). These inequalities in obesity can contribute to social disparities in chronic disease mortality (Hoffmann et al., 2015).

Built environment attributes of neighbourhoods are also social determinants of health and can influence residents' obesity risk. For instance, higher walkability, lower urban sprawl (Chandrabose et al., 2019a) and the presence of walkable destinations (Yamada et al., 2012) are associated with a lower risk of obesity. However, such health-promoting built environmental attributes are also unevenly distributed within society (Bernard et al., 2007). Given that neighbourhoods vary both in socioeconomic and built environmental attributes, the disparity in obesity between lower- and higher-SES areas may differ depending on local environmental attributes. A recent review on this topic identified nine studies (Selvakumaran et al., 2023), most of which reported non-significant effect modification of the relationship between area-level SES and obesity by environmental attributes. A few studies found moderation by attributes such as urbanicity (Feuillet et al., 2020), aesthetics and safety (Oyeyemi et al., 2012), with mixed findings for walkability (Sallis et al., 2009; McCormack et al., 2017). Given the limited and mixed findings, further research is needed to understand the role of built environmental attributes in socioeconomic inequalities in obesity.

Population density is a fundamental built environmental attribute as it can influence various aspects of neighbourhood-level urban form (Fonseca et al., 2022). For instance, low density areas tend to have poor access to shops, services, recreational facilities and public transport options, which can limit physical activity opportunities (Giles-Corti et al., 2012). A cross-sectional study conducted in Australia identified that living in low density areas was associated with a higher risk of obesity (Garden and Jalaludin, 2009), and similar findings have been also reported in the USA (Lopez, 2007). These findings suggest that population density, which differs substantially across neighbourhoods, can be a factor affecting the distribution of obesity.

Despite such a possibility, little is known about whether population density can moderate the associations of area-level SES with obesity. One cross-sectional study conducted in Portugal examined this issue but found a non-significant interaction effect between area-level SES and population density on obesity, i.e., no evidence of moderation (Santana et al., 2009). There are several potential reasons for such findings. This study used body mass index (BMI) as the measure of obesity based on self-reported height and weight, which can be subject to bias (Gugushvili and Jarosz, 2019). A measure of deprivation used in the study consisted of social cohesion, diverse local resources and public health services. This is different from a typical measure of area-level SES, which commonly includes variables related to income, education and occupation. Since the study was conducted in a single city (Lisbon), there may have also been limited variability in population density.

Another gap identified in the above-mentioned review is that most studies on this topic have been cross-sectional in design (Selvakumaran et al., 2023). Given evidence of widening socioeconomic gaps in obesity over time (Feng and Wilson, 2015; Powell-Wiley et al., 2014), it is important to explore the potential role of environmental attributes in reducing or increasing socioeconomic inequalities in obesity.

We examined whether neighbourhood population density moderated the association of area-level SES with waist circumference and its 12-year change in a cohort of Australian adults.

## 2 Methods

### 2.1 Data Source

The study used data from the Australian Diabetes, Obesity, Lifestyle (AusDiab) study, a national longitudinal study designed to investigate diabetes and associated risk factors among Australian adults (Dunstan et al., 2002). Data were collected in three waves in 1999–2000, 2004–2005 and 2011–2012. At baseline, participants were recruited using a two-stage stratified cluster sampling method from 42 randomly chosen study sites across the six states and the Northern Territory. Each study site comprised one to four adjoining census collection districts (CCDs), the smallest geographic unit (with an average of 225 dwellings) at the time of baseline (Australian Bureau of Statistics, 2001a). We excluded study sites in remote or regional areas (*n* = 22) because the study sought to compare socioeconomic inequalities in obesity between different levels of population density. If remote/regional areas, typically low in population density, are included, the comparison would be confounded by differences between urban and rural contexts, which is a distinct research topic. Ethics approval for the study was granted by the Alfred Hospital Ethics Committee (no. 39/11). Written informed consent was obtained from participants.

### 2.2 Study Participants

Cross-sectional sample: At baseline, 11247 adults aged 25 years and older were recruited. As mentioned above, participants living in remote or regional areas (*n* = 5824) were excluded. Those who had missing values for the variables used (*n* = 499) or were pregnant at baseline (*n* = 89) were also excluded. The final cross-sectional sample size was 4835.

Longitudinal sample: A total of 4614 participants provided 12-year follow-up data. Of them, 2369 participants stayed in the same residence throughout the study. We focused on those who stayed in the same address because this study investigated the moderating role of baseline population density. Examining the change in population density due to relocation would be a complex question, requiring a separate investigation. Those who resided in regional or remote areas (*n* = 1181), along with those who had missing values for the variables used (*n* = 15) or were pregnant (n = 15) were excluded. The final longitudinal sample size was 1158.

### 2.3 Measures

Outcomes: Participant's waist circumference was measured at each wave by trained personnel (Dunstan et al., 2002). Waist circumference was chosen as the outcome of the study since it is a proxy measure of abdominal adiposity and a stronger predictor of all-cause and cardiovascular mortality than BMI (Ross et al., 2020). It is particularly relevant in longitudinal research as it can measure changes in fat distribution that can occur independent of weight gain (Gearon et al., 2018). For cross-sectional analysis, we used baseline waist circumference as the outcome. For longitudinal analysis, we used the rate of waist circumference change over 12 years, which was calculated using linear growth models (see Data Analysis for details).

Exposure: Area-level SES at baseline was used as the exposure variable in both cross-sectional and longitudinal analyses. This was measured at the CCD level using the 2001 Index of Relative Socio-economic Disadvantage (IRSD), which is a census-based index consisting of 16 area-level disadvantage measures, such as the percentage of low-income households and unemployed people (Australian Bureau of Statistics, 2001b). This commonly used indicator of neighbourhood-level disadvantage is used to assess inequalities in health in Australia (Australian Institute of Health and Welfare, 2016). The IRSD has a mean of 1000 (the national average) and a standard deviation of 100, with lower scores indicating greater disadvantage (Australian Bureau of Statistics, 2001b). In each study site, the IRSD score was calculated by aggregating the score of each member CCD with its population as the weight.

Potential moderator: Baseline population density was measured at the individual level, by dividing the population count within a 1 km radius circular buffer around each participant's residence by its total area and expressed as person/ha, as previously detailed in another Australian study (Chandrabose et al., 2019b). A 1 km buffer is commonly used to represent a neighbourhood area in previous studies on health and place (Adams et al., 2014). We did not use the population density of study sites since they differed greatly in geographic size (median size = 0.29 km^2^, range: 0.09–6.11 km^2^). The population count data was obtained from 2001 census data for each CCD (Australian Bureau of Statistics, 2001c).

### 2.4 Data Analysis

#### 2.4.1 Cross-sectional analysis

We used a two-level linear regression model (level 1: participants; level 2: study sites) to assess the association between the IRSD and waist circumference at baseline. IRSD was used as a continuous variable, with one unit corresponding to the national standard deviation of 100. This model included a random intercept to account for the clustering of participants within study sites and was adjusted for age, gender, employment status, education level, and household income as covariates. To test the interaction effect of population density and IRSD on waist circumference, we added an interaction term to this regression model. We divided the sample into tertiles based on baseline population density (lower, medium, and higher) and conducted stratified analyses by fitting a separate regression model for each tertile.

#### 2.4.2 Longitudinal analysis

We fitted an unconditional two-level linear growth model (Hox et al., 2017) first to obtain participant's annual change in waist circumference. For each participant, waist circumference values were modelled with time metrics: t1 = 0 for baseline, t2 = 5 for the 5-year follow-up, and t3 = 12 for the 12-year follow-up, and the model included random slopes for time, which were allowed to vary between participants. The estimated random slopes represent the annual rate of change in waist circumference for each participant (cm/year). This approach, instead of simply subtracting the baseline value from the last follow-up, allowed us to use all three data points collected during the study for a more accurate assessment of change in the outcome measure. To examine the association between baseline IRSD and the rate of change in waist circumference, we used a three-level linear growth model. Time was specified as level 1, nested within individual participants (level 2), who were clustered within study sites (level 3). The model was adjusted for the baseline age, gender, employment status, education level, household income, and follow-up period. To assess the interaction between baseline IRSD and population density on the rate of change in waist circumference, we included a three-way interaction term in the model. Then, we fitted three separate regression models within each population density tertile All statistical analyses were conducted using R version 4.2.1 and the ‘lme4’ package was used to conduct the regression analysis and the growth models.

## 3 Results

Table 1 shows the characteristics of the cross-sectional and longitudinal samples. The mean baseline waist circumference was 90 cm for the cross-sectional sample. This was slightly reduced for the longitudinal sample due to attrition. The unconditional linear growth model estimated that participants on average increased their waist circumference by 0.47 cm/year (SD 0.19). In the cross-sectional sample, household income, employment, and education data were missing for 1.4 %, 12.6 %, and 0.6 % of participants, respectively. In the longitudinal sample, missing values for these variables were 2.1 %, 0.7 %, and 0.3 %.

**Table 1:** Characteristics of the cross-sectional [1999/2000] and longitudinal [1999/2000–2011/2012] samples of adults from the Australian Diabetes, Obesity, Lifestyle study.

	N [%] or Mean (SD)	
	Cross-sectional	Longitudinal
N (participants)	4835	1158
Baseline age	52.5 (14.5)	51.2 (10.8)
Gender, women	2643 [54.7]	614 [53.0]
Education status		
High school or less	1777 [36.8]	357 [30.8]
Technical or vocational	1985 [41.1]	495 [42.7]
Bachelor's degree or more	1045 [21.6]	302 [26.1]
Employment status, working	2783 [57.6]	815 [70.4]
Household income		
<$600 pw	1954 [40.4]	311 [26.9]
$600–1500 pw	1894 [39.2]	494 [42.7]
>$1500 pw	917 [19.0]	329 [28.4]
Not reported	70 [1.4]	24 [2.1]
Follow-up period, years	–	11.9 (0.3)
Baseline waist circumference, cm	90.0 (13.7)	89.1 (13.4)
12-year change in waist circumference, cm	–	5.7 (7.2)
Baseline population density, persons/ha	18.1 (8.3)	17.0 (7.8)

Supplementary Table 1 presents characteristics of the 20 study sites (CCD clusters) across five Australian states, detailing the number of CCDs, area size, population, IRSD score, and participant counts for both cross-sectional and longitudinal samples. Study site areas ranged from 0.18 to 10.95 km^2^, populations from 550 to 2265, and IRSD scores reflected a range of socioeconomic advantage and disadvantage. Supplementary Table 2 shows correlation coefficients between variables. In the cross-sectional sample, IRSD had a weak negative correlation with waist circumference and population density. However, no significant correlations were observed between waist circumference and population density. These correlation patterns are similar in the longitudinal sample.

Table 2 shows the main effects of area-level SES and interaction effects with population density on waist circumference measures. The cross-sectional main effect model found a significant negative association between area-level SES and waist circumference. One SD (=100) higher IRSD score (i.e., greater advantage) was associated with a 1.45 cm lower waist circumference. The longitudinal main effect model found no significant association between area-level SES and the rate of waist circumference change. A significant interaction was observed between area-level SES and lower and medium density areas (compared to higher density areas) on baseline waist circumference. Similarly, significant interactions were observed between area-level SES and both lower and medium density areas (compared to higher density areas) on the rate of waist circumference change.

**Table 2:** Effects of area-level socioeconomic status (Index of Relative Socioeconomic Disadvantage, IRSD) and its interaction with population density on waist circumference outcomes for a sample of adults from the Australian Diabetes, Obesity, Lifestyle study.

Outcomes	B (95 % CI)			
	Main effect of IRSD⁎	Interaction effects between IRSD and density tertiles⁎⁎		
		IRSD x Lower density	IRSD x Medium density	IRSD x Higher density
Baseline waist circumference (Cross-sectional analysis [1999/2000], *n* = 4835)	−1.45 (−2.78, −0.12)	−2.81 (−5.34, −0.28)	−1.50 (−3.03, −0.03)	Ref
Rate of waist circumference change (Longitudinal analysis [1999/2000–2011/2012], *n* = 1158)	0.04 (−0.02, 0.09)	0.24 (0.09, 0.38)	0.25 (0.10, 0.40)	Ref

All models used IRSD as a continuous variable with one unit corresponding to the national standard deviation of 100. Models for waist circumference adjusted for age, gender, employment status, education status and household income. Models for waist circumference change further adjusted for follow-up period. All models were corrected for area-level clustering.

⁎ Models examining the main effects of IRSD on waist circumference were fitted without the interaction term.

⁎⁎ Interaction models display the coefficients for the interaction between IRSD and population density tertiles.

Table 3 shows the association of area-level SES with baseline waist circumference and with the rate of waist circumference change, stratified by population density tertiles. Characteristics of the samples for lower, medium and higher density tertiles are shown in Supplementary Table 3. Although differences related to waist circumference between the tertiles were modest, higher density areas had the highest baseline values, but the smallest 12-year change and rate of change compared to lower and medium density areas. The association between area-level SES and waist circumference was significant in lower density and medium density areas but not in higher density areas. In the longitudinal sample, the association between area-level SES and rate of waist circumference change was significant across all tertiles of population density. However, the direction of the associations differed between them. It was positive in lower density and medium density areas, where a higher IRSD score (greater advantage) was associated with a greater increase in waist circumference. However, the association was negative in higher density areas, where a higher IRSD score was associated with a smaller increase in waist circumference.

**Table 3:** Association of area-level socioeconomic status (Index of Relative Socioeconomic Status, IRSD) with baseline waist circumference and rate of waist circumference change, stratified by population density tertiles for a sample of adults from the Australian Diabetes, Obesity, Lifestyle study.

Outcomes	Lower density [0.4–15.3 persons/ha]	Medium density [15.4–21.0 persons/ha]	Higher density [21.1–52.3 persons/ha]
	B (95 % CI)	B (95 % CI)	B (95 % CI)
Baseline waist circumference [1999/2000], cm	−5.00 (−7.73, −2.28)	−2.61 (−4.55, −0.67)	−0.54 (−1.73, 0.65)
	Lower density [0.4–14.1 persons/ha]	Medium density [14.2–19.8 persons/ha]	Higher density [19.9–52.3 persons/ha]
Rate of waist circumference change [1999/2000–2011/2012], cm/year	0.16 (0.03, 0.29)	0.17 (0.05, 0.30)	−0.08 (−0.15, −0.00)

Models for waist circumference adjusted for age, gender, employment status, education status and household income. Model for waist circumference change also adjusted for follow-up period. All models were corrected for area-level clustering. Regression coefficients correspond to one SD (100) increment in IRSD (Index of Relative Socioeconomic Disadvantage) score.

## 4 Discussion

We found a significant socioeconomic gradient in obesity at baseline (i.e., lower area-level SES associated with larger waist circumference), which is in line with previous Australian and international research (Mohammed et al., 2019; Carroll et al., 2023). In analyses stratified by population density, socioeconomic inequalities in obesity remained significant in areas with lower and medium population density, with the largest socioeconomic gradient observed in lower density areas. This suggests that residents of low SES areas may be more susceptible to the environmental constraints provided by lower population density, such as poor access to local shops, services, and transport stops. Residents of high SES areas may have additional means/resources to cope with such constraints. It is also possible that lower density lower-SES and lower density higher-SES areas may differ in some key environmental features, such as the availability of recreational facilities (e.g., parks, gyms, and sports grounds). On the other hand, we found non-significant socioeconomic gradient in obesity in higher density areas. It can be argued based on the results that high density environments, which tend to be more walkable, may be equally beneficial to residents of low and high SES neighbourhoods.

In longitudinal analysis, we found that participants on average increased their waist circumference by approximately 0.5 cm annually, but this change did not differ by neighbourhood SES levels. Although there was a lack of significant association between area-level SES and waist circumference change for the whole sample, different patterns of association emerged in the analyses stratified by population density tertile. In lower and medium density areas, the association of area-level SES with waist circumference change was positive, i.e., a greater increase in waist circumference in higher-SES areas. However, the opposite was the case in higher density areas (i.e., lower-SES associated with a greater increase). This suggests that SES-related inequalities in obesity, which were observed in low and medium density areas at baseline, seemed to be decreasing during the study period. However, in higher density areas, where socioeconomic disparities in obesity were not observed at baseline, disparities seemed to be growing over time. Making sense of these findings is challenging. One possibility is regression to the mean, the statistical phenomenon where follow-up measurements are more likely to be closer to the population average (Allison et al., 2009). According to this notion, residents of lower density, lower-SES areas, who tended to have a higher waist circumference at baseline, would be less likely to experience a larger increase in waist circumference at follow-up. An alternative explanation for the findings may be derived from health behaviours, which vary by socioeconomic status and population density. For instance, it has been shown that socioeconomic status and walkability (in which population density is a key component) interact in affecting dietary patterns: higher density was related to health-enhancing dietary behaviours in advantaged areas, but the opposite was the case in disadvantaged areas (Chandrabose et al., 2022). Physical activity is also known to be related to socioeconomic status and environmental attributes in a complex manner (Sugiyama et al., 2015). It is possible that residents of areas with differing levels of disadvantage and density may engage in different health behaviour patterns, which could accumulate over time to contribute to changes in waist circumference.

Although the findings of longitudinal moderation are difficult to interpret, the effect size shown in Table 3 seems to suggest that the socioeconomic disparity in waist circumference in lower density area at baseline may not be alleviated in a short period of time. At baseline, one SD higher IRSD score was associated with 5 cm lower waist circumference. The same difference in IRSD score was associated with 0.16 cm higher waist circumference increase per year. This means that disparities in waist circumference would remain even after 10 years (1.6 cm increase) in areas with lower population density. Our findings suggest that socioeconomic inequalities in obesity would be persistent in low density areas.

Our study identified that the threshold between low/medium and higher density area was around 20 person/ha in this sample (Supplementary Table 3). This means that developing suburbs with a density lower than this threshold may contribute to greater socioeconomic inequalities in obesity. Considering that two thirds of the study participants resided in areas with density lower than this, it may not be practical to set this level of population density as a target in the context of Australia. However, it should be noted that this density level is much lower than the density target of 15 dwellings/ha set by municipalities such as Sydney, Melbourne and Brisbane (Arundel et al., 2017). The threshold identified in this study is equivalent to 8 dwellings/ha, with the average household size of 2.5 persons (Australian Bureau of Statistics, 2001d). The density level obtained in this study may be employed as a more feasible (and evidence-based) density target for future urban residential developments.

Strengths of this study include a longitudinal design (with 5-year and 12-year follow-up data), employing an objective measure of waist circumference, and use of data collected from diverse urban settings in Australia. Limitations of the study include that Australia is generally low in population density. While 89 % of Australians live in cities, most reside in low to medium density areas, with cities tend to encompass sprawling outer suburbs (Feng and Wilson, 2017a). In contrast, Asian and European cities are higher in density with better access to relevant destinations such as shops, services and public transport (Kenworthy, 2017; Spencer et al., 2015). These structural differences may impact the generalisability of our findings to high density urban settings. Future research should investigate whether socioeconomic inequalities in obesity are smaller in higher density neighbourhoods in diverse settings. Another limitation of the study is that the longitudinal sample was reduced considerably partly due to attrition of participants during the study period. Although it is highly common to lose participants in longitudinal studies, our findings may be affected by systematic attrition. Future research needs to understand the pathways through which higher density can mitigate socioeconomic inequalities in waist circumference. Potential pathways include other built environment features (e.g., healthy food access, walkability, proximity to facilities), behavioural factors (physical activity, diet), and psychosocial factors (stress, social cohesion). Investigating such pathways can explain how characteristics of high-density environments contribute to reducing inequalities. Future studies may focus on women in understanding the moderating effects of population density, as research suggests that the socioeconomic gradient in obesity is steeper in women (Feng and Wilson, 2017b). Findings from such research can inform strategies to reduce socioeconomic inequalities in obesity among women, which is likely to be an effective strategy to achieve equity.

## 5 Conclusion

Our study found significant socioeconomic inequalities in obesity in lower and medium density areas, where lower-SES was associated with higher baseline waist circumference. In contrast, such inequalities were not observed in higher density areas. The longitudinal findings suggest that these inequalities may decrease over time in lower and medium density areas but grow in higher density areas. However, since these annual changes were small, the inequalities are likely to persist in lower density areas. Our findings suggest that creating low density residential neighbourhoods, which are often developed for low-income households, can widen socioeconomic inequalities in obesity. The urban planning and public health sectors need to work together to promote building higher density neighbourhoods or increasing the density of existing neighbourhoods to address socioeconomic inequalities in obesity.

## CRediT authorship contribution statement

**Sungkavi Selvakumaran:** Writing – original draft, Formal analysis, Conceptualization. **Manoj Chandrabose:** Writing – review & editing, Methodology, Conceptualization. **Nyssa Hadgraft:** Writing – review & editing, Conceptualization. **Neville Owen:** Writing – review & editing, Methodology, Conceptualization. **Takemi Sugiyama:** Writing – review & editing, Methodology, Conceptualization.

## Funding sources

SS is supported by the Swinburne University Postgraduate Research Award (SUPRA) scholarship by Swinburne University of Technology. MC is supported by the ARC-funded discovery project “Designing Liveable Neighbourhoods for Healthy Ageing” (#DP230102101). The funders had no role in study design, data collection and analysis, decision to publish, or preparation of the manuscript.

## Declaration of competing interest

The authors declare that they have no known competing financial interests or personal relationships that could have appeared to influence the work reported in this paper.

## Data Availability

The authors do not have permission to share data.

## Appendix A Supplementary data

Supplementary Table 1: Characteristics of study sites for the Australian Diabetes, Obesity, Lifestyle study; Supplementary Table 2. Pearson’s correlation coefficients between key measures for a sample of adults from the Australian Diabetes, Obesity Lifestyle study; Supplementary Table 3. Characteristics of the cross-sectional (1999/2000) and longitudinal samples (1999/2000 – 2011/2012) by population density tertiles for a sample of adults from the Australian Diabetes, Obesity, Lifestyle study. Image 1
