# Supplementary material for: Socioeconomic inequalities in waist circumference and its 12-year change: Moderation by neighbourhood population density
Source: Prev Med Rep. 2025 Sep 11;59:103236. doi: 10.1016/j.pmedr.2025.103236 (PMC12478090; doi:10.1016/j.pmedr.2025.103236)
Supplement: Supplementary file 1 — Supplementary Table 1: Characteristics of study sites for the Australian Diabetes, Obesity, Lifestyle study; Supplementary Table 2. Pearson’s correlation coefficients between key measures for a sample of adults from the Australian Diabetes, Obesity Lifestyle study; Supplementary Table 3. Characteristics of the cross-sectional (1999/2000) and longitudinal samples (1999/2000 – 2011/2012) by population density tertiles for a sample of adults from the Australian Diabetes, Obesity, Lifestyle study. [file mmc1.docx]

**SUPPLEMENTARY TABLES**

Supplementary Table 1: Characteristics of study sites for the Australian Diabetes, Obesity, Lifestyle study.

| **Site Name** | **State** | **# of CCDs** | **Area (km^2^)** | **Population** | **IRSD* Score** | **Cross-sectional Sample size (n)** | **Longitudinal Sample size (n)** |
| --- | --- | --- | --- | --- | --- | --- | --- |
| Parkdale | Victoria | 1 | 0.18 | 550 | 1090 | 183 | 60 |
| Blackburn | Victoria | 1 | 0.2 | 578 | 1074 | 216 | 44 |
| East Burwood | Victoria | 1 | 0.29 | 768 | 1036 | 154 | 43 |
| Wattle Glen | Victoria | 2 | 10.95 | 701 | 1078 | 225 | 76 |
| Scarborough | Western Australia | 3 | 0.47 | 1150 | 1059 | 217 | 48 |
| Trigg | Western Australia | 3 | 1.47 | 618 | 1076 | 179 | 27 |
| Kardinya | Western Australia | 3 | 1.13 | 2000 | 1069 | 361 | 97 |
| High Wycombe | Western Australia | 3 | 5.35 | 2137 | 1028 | 201 | 25 |
| West Penant Hills | New South Wales | 2 | 2.35 | 2265 | 1133 | 307 | 112 |
| Hurstville | New South Wales | 3 | 0.56 | 2147 | 989 | 191 | 25 |
| Auburn | New South Wales | 3 | 1.22 | 1947 | 874 | 105 | 10 |
| Grays Point | New South Wales | 2 | 0.76 | 1398 | 1121 | 254 | 104 |
| Berkeley Vale | New South Wales | 3 | 3.94 | 2047 | 1019 | 296 | 63 |
| Hyde Park | South Australia | 3 | 0.65 | 1647 | 1127 | 250 | 69 |
| North Plympton | South Australia | 3 | 0.95 | 1930 | 1002 | 345 | 96 |
| Glenelg | South Australia | 3 | 0.42 | 1398 | 1083 | 287 | 65 |
| Parafield Gardens | South Australia | 2 | 0.78 | 1795 | 817 | 221 | 32 |
| Stafford Heights | Queensland | 2 | 0.82 | 1669 | 983 | 304 | 57 |
| Chapel Hill | Queensland | 2 | 0.7 | 1517 | 1121 | 255 | 69 |
| Currumbin | Queensland | 2 | 0.54 | 1252 | 1006 | 284 | 36 |

*Index of Relative Socioeconomic Disadvantage

Supplementary Table 2. Pearson’s correlation coefficients between key measures for a sample of adults from the Australian Diabetes, Obesity Lifestyle study.

|  | 1 | 2 | 3 | 4 |
| --- | --- | --- | --- | --- |
| 1. Baseline IRSD score | — | -0.11*** | 0.03 | -0.25*** |
| 2. Baseline waist circumference | -0.09*** | — | -0.04 | 0.03 |
| 3. Rate of waist circumference change | . | . | — | -0.04 |
| 4. Baseline population density | -0.27*** | 0.01 | . | — |

* p < .05, ** p < .01, *** p < .001

Bottom left: Cross-sectional sample (1999/2000, N= 4,835)

Upper right: Longitudinal sample (1999/2000 – 2011/2012, N= 1,158)

Supplementary Table 3. Characteristics of the cross-sectional (1999/2000) and longitudinal samples (1999/2000 – 2011/2012) by population density tertiles for a sample of adults from the Australian Diabetes, Obesity, Lifestyle study.

|  | N, Mean (SD), Median or % | | | | | | | |
| --- | --- | --- | --- | --- | --- | --- | --- | --- |
|  | **Cross-sectional** | | |  | **Longitudinal** | | |  |
|  | Lower  density | Medium density | Higher density |  | Lower  density | Medium density | Higher density |  |
| N (participants) | 1,612 | 1,612 | 1,611 |  | 386 | 386 | 386 |  |
| Baseline age | 51.3 (13.5) | 53.7 (14.8) | 52.5 (14.9) |  | 49.9 (9.88) | 51.6 (10.9) | 52.2 (11.6) |  |
| Gender, women | 883 (54.8%) | 871 (54.0%) | 889 (55.2%) |  | 213 (55.2%) | 201 (52.1%) | 200 (51.8%) |  |
| Baseline employment status, working | 1022 (63.4%) | 893 (55.4%) | 868 (53.9%) |  | 294 (76.2%) | 272 (70.5%) | 249 (64.5%) |  |
| Baseline household income, <$600pw | 535 (33.2%) | 678 (42.1%) | 741 (46.0%) |  | 86 (22.3%) | 103 (26.7%) | 140 (36.3%) |  |
| Baseline household income, $600-$1500pw | 665 (41.3%) | 620 (38.5%) | 609 (37.8%) |  | 178 (46.1%) | 160 (41.5%) | 156 (40.4%) |  |
| Baseline household income, >$1500pw | 382 (23.7%) | 291 (18.1%) | 244 (15.1%) |  | 112 (29.0%) | 114 (29.5%) | 85 (22.0%) |  |
| Baseline education, high school or less | 573 (35.5%) | 570 (35.4%) | 634 (39.4%) |  | 118 (30.6%) | 107 (27.7%) | 132 (34.2%) |  |
| Baseline education, technical or vocational | 703 (43.6%) | 686 (42.6%) | 596 (37.0%) |  | 179 (46.4%) | 164 (42.5%) | 152 (39.4%) |  |
| Baseline education, bachelor’s degree or more | 325 (20.2%) | 351 (21.8%) | 369 (22.9%) |  | 87 (22.5%) | 114 (29.5%) | 101 (26.2%) |  |
| Baseline waist circumference, cm | 89.6 (13.8) | 89.8 (13.4) | 90.5 (13.9) |  | 88.2 (13.1) | 88.8 (13.5) | 90.4 (13.7) |  |
| 12-year change in waist circumference, cm | — | — | — |  | 6.56 (7.26) | 5.50 (6.67) | 5.06 (7.63) |  |
| Rate of waist circumference change, cm/year | — | — | — |  | 0.50 (0.19) | 0.47 (0.17) | 0.46 (0.20) |  |
| Baseline population density, persons/ha | 9.9 (4.4) | 18.1 (1.6) | 26.4 (6.8) |  | 8.9 (4.2) | 17.0 (1.6) | 25.0 (5.7) |  |
| Median baseline population density [min, max], persons/ha | 11.0  [0.4, 15.3] | 18.3  [15.4, 21.0] | 24.4  [21.1, 52.3] |  | 10.2  [0.4, 14.1] | 17.0  [14.2, 19.8] | 23.9  [19.9 52.3] |  |
